# Supplementary material for: In vitro maturation of Toxoplasma gondii bradyzoites in human myotubes and their metabolomic characterization
Source: Nat Commun. 2022 Mar 4;13:1168. doi: 10.1038/s41467-022-28730-w (PMC8897399; doi:10.1038/s41467-022-28730-w)
Supplement: Supplementary file 1 — Supplementary Information [file 41467_2022_28730_MOESM1_ESM.pdf]

Fig. S1

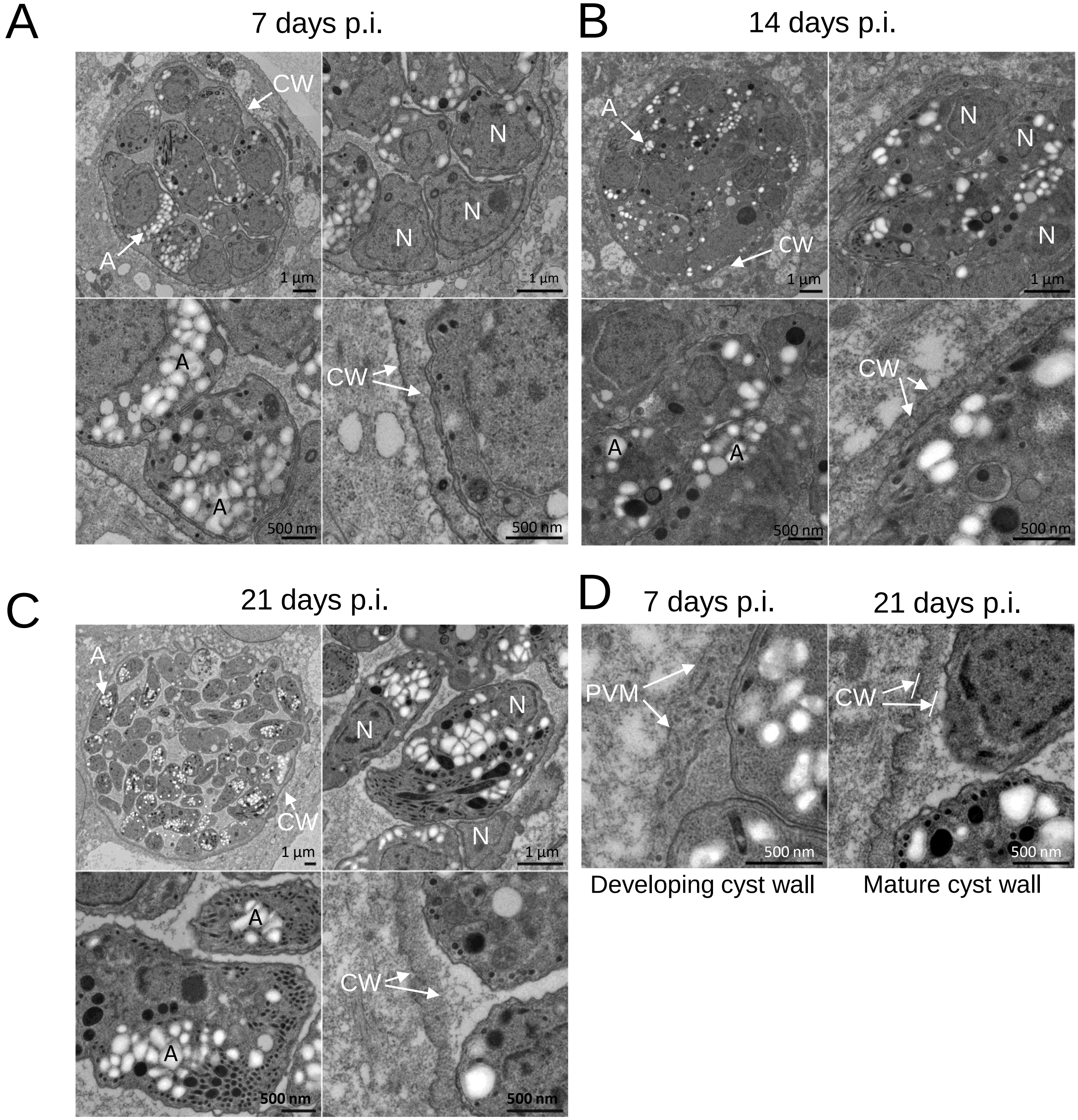

Figure S1: Shown are micrographs of maturing tissue cysts Pru-tdTomato parasites in KD3 human myotubes. Electron microscopy of (A.) 7-, (B.) 14- and (C.) 21-day-old Pru-tdTomato tissue cysts matured in KD3 myotubes as shown in Figure 2 (p.i.: post infection). Images of a single section plane through entire cysts were taken for each time point. Each overview image (upper left) is grouped with three images showing enlarged areas, such as individual parasites with their nuclei (N), amylopectin granules (A) or the cyst wall (CW). In the example shown here, the cyst wall is already well developed 7 days post infection. Other cysts at this time point reveal an immature cyst wall, which is shown in the left image of the additional image panel on the lower right side (D.). At this stage the parasitophorous vacuolar membrane (PVM) is associated with a loose matrix containing vesicles and membrane-bound tubules. In cysts 21 days post induction (shown at the right side of this image panel), the cyst wall (CW) is formed by a dense and rather homogenous matrix, which is associated with the PVM. Images are representative of at least two independent experiments.

Fig. S2

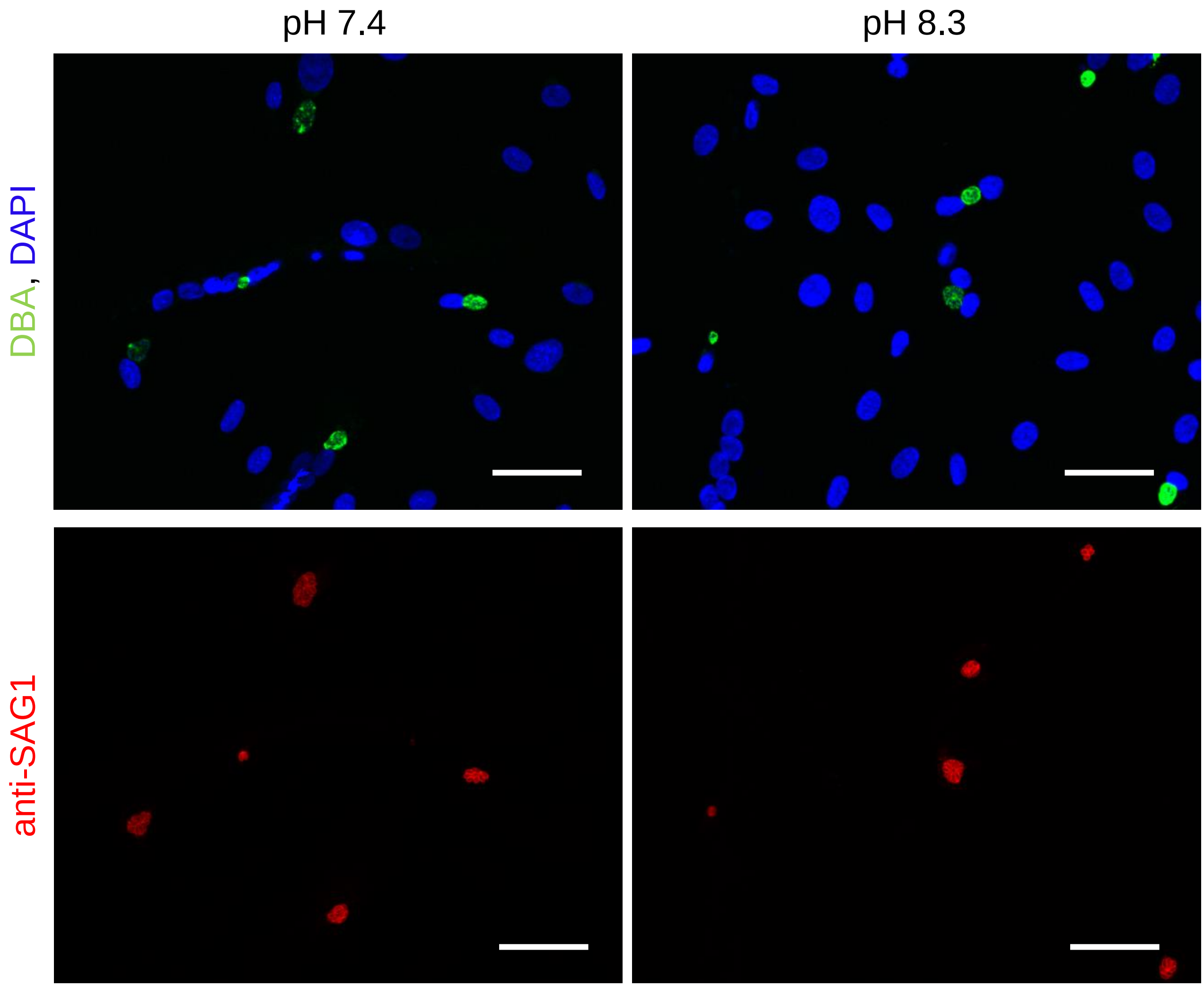

Figure S2: Immunofluorescence imaging of KD3 myotubes infected with type I RHΔ*ku80*. Cyst formation was induced for 7, 14 and 21 days under neutral or basic pH and stained with anti-SAG1 antibodies, DBA and DAPI. Shown are representative images of 21-day-old RHΔ*ku80* tissue cysts cultured in neutral (left image) and cultured in basal pH (right image). Scale bar indicates 50 µm. Images are representative of experiments shown in figure 2.

Fig. S3

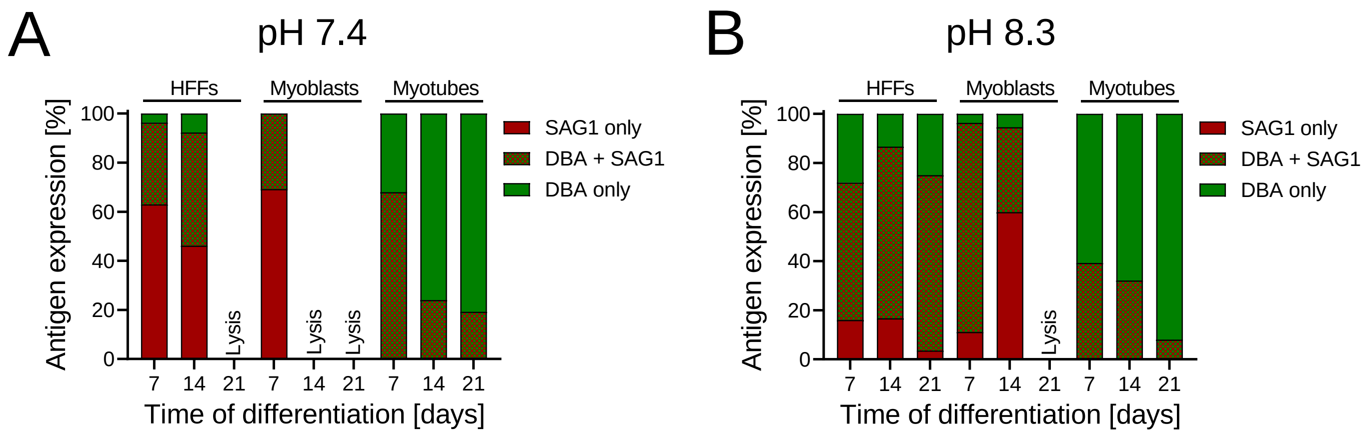

Figure S3: Time- and pH-dependent maturation of tissue cysts of type II Pru-tdTomato parasites in human fibroblasts, human KD3 myoblasts and myotubes. (A.-B.) Cyst formation was induced for 7, 14 and 21 days in human fibroblasts, KD3 myoblasts and myotubes under neutral (A.) or basic (B.) pH and stained with anti-SAG1 antibodies, DBA and DAPI. Displayed are percentage of vacuoles that only expressed SAG1 but not DBA, vacuoles that co-expressed SAG1 and DBA and vacuoles that exclusively expressed DBA but lacked SAG1. Values represent means from one blinded experiment. At least 25 images were taken and processed per cell line, time point and pH. Source data are provided as a Source Data file.

Fig. S4

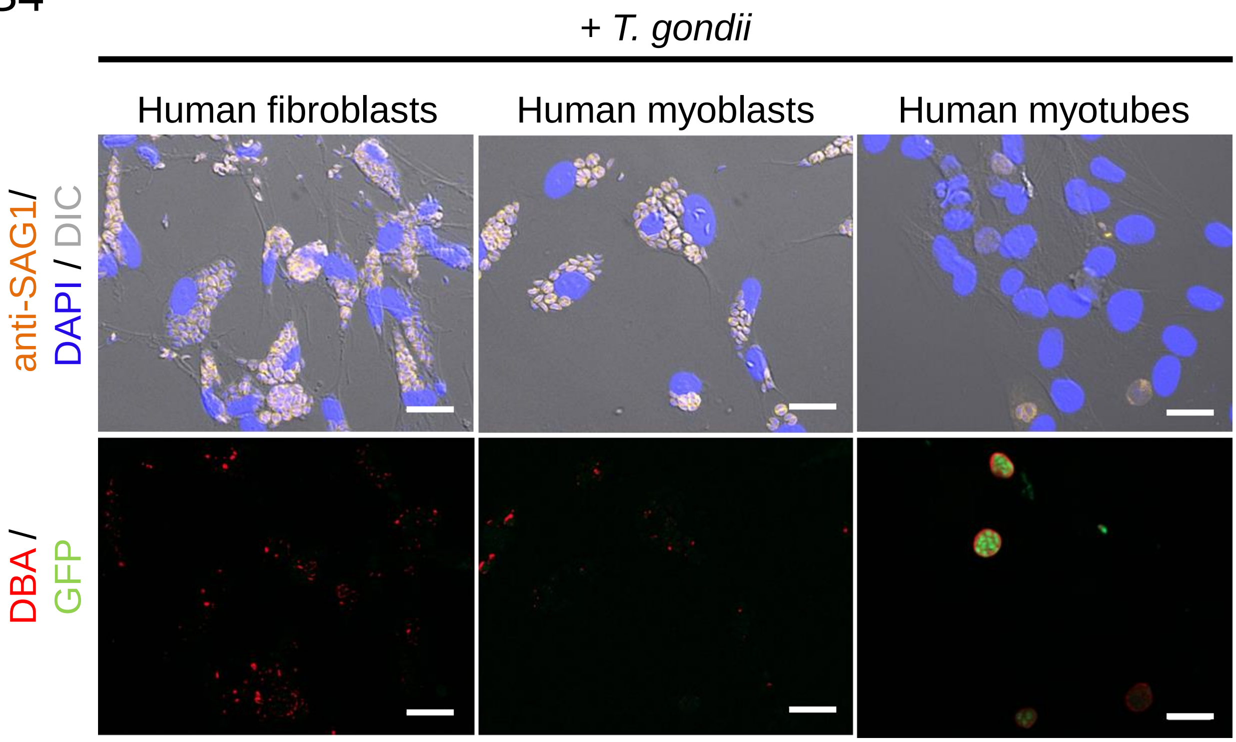

Figure S4: Influence of different host cell environments on spontaneous cystogenesis of *T. gondii*. Human KD3 myoblasts, myotubes and fibroblasts were infected with *T. gondii* Pru-GFP that express GFP under the bradyzoite-specific LDH2-promoter for 96 h. Monolayers were stained with anti-SAG1 antibodies, DBA and DAPI. The GFP signal indicates spontaneous stage conversion to early bradyzoite stages 96 h post-infection in bicarbonate-replete conditions. Scale bar indicates 20 µm. Images are representative of one experiment.

Fig. S5

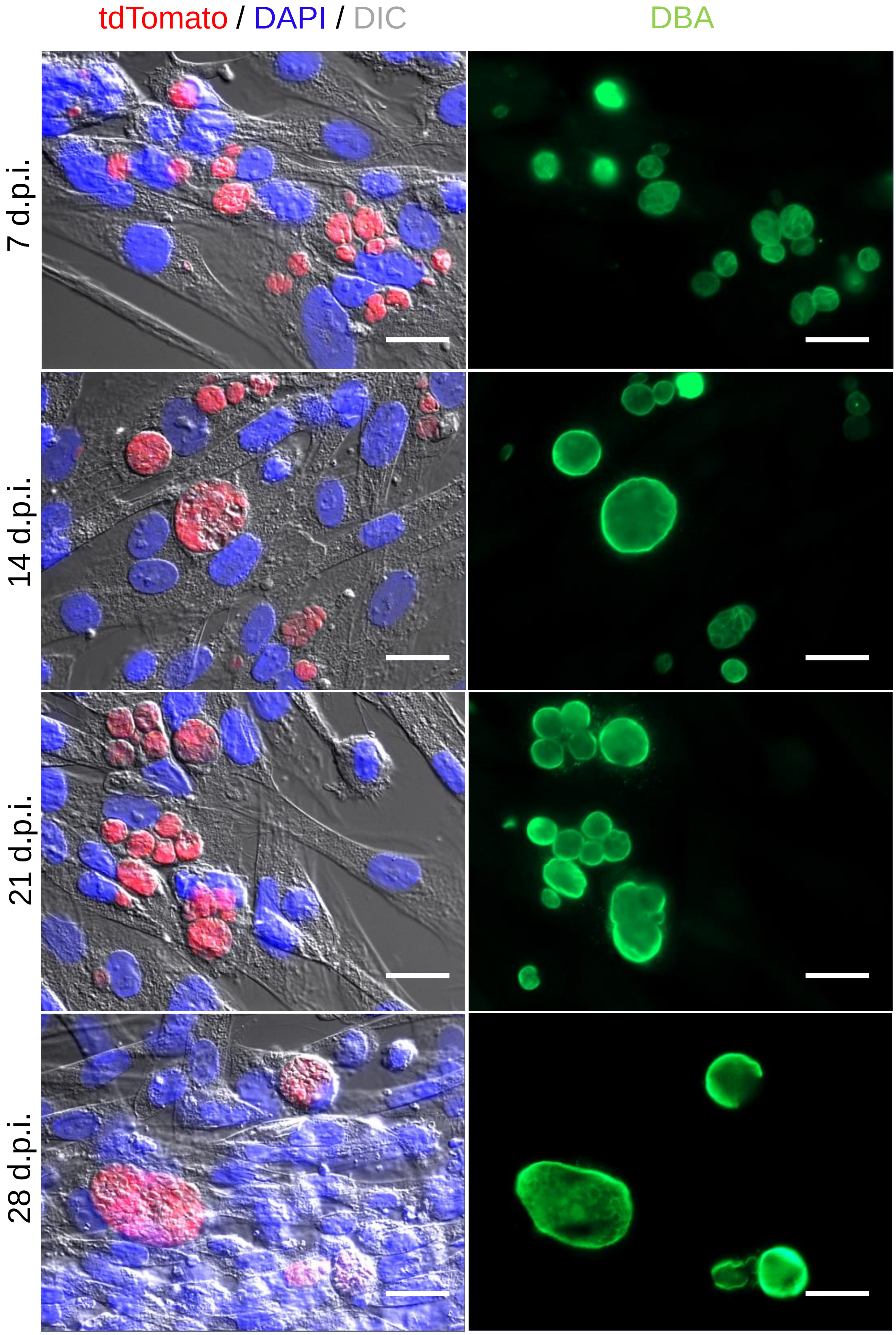

Figure S5: Immunofluorescence imaging of maturing encysted bradyzoites of Pru-tdTomato parasites in KD3 myotubes. Human KD3 myotubes were infected with Pru-tdTomato tachyzoites under bradyzoite inducing conditions for indicated times and stained with DBA and DAPI. Shown are representative images of 7-, 14-, 21- and 28-day-old Pru-tdTomato tissue cysts. Scale bar indicates 20 µm. Representative of one experiment (d.p.i. days post infection).

Fig. S6

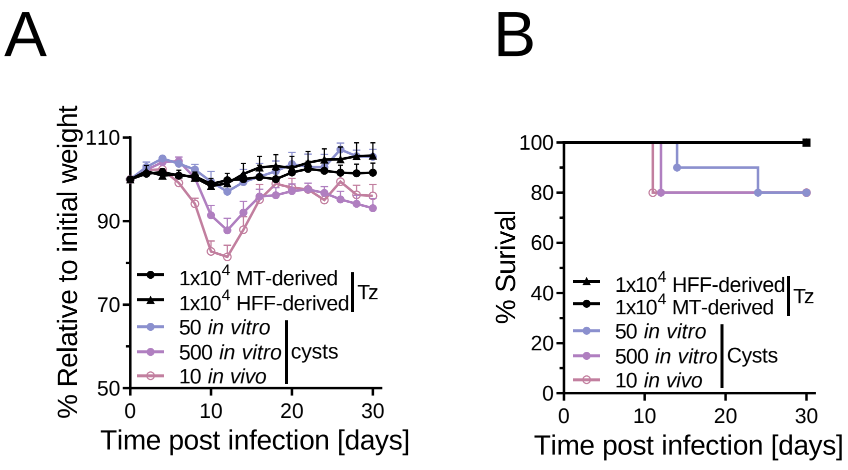

Fig S6: Weight loss and survival of mice infected with Pru-tdTomato tachyzoites and tissue cysts. (A.-B.) Mice were orally infected with either 1x10^4 myotube (MT)- or human fibroblast (HFF)-derived tachyzoites, 50 or 500 *in vitro*-generated or 10 *in vivo*-generated tissue cysts for 30 days. (A.) The relative weight loss displayed in %. Each data point represents the means of two experiments consisting of five infected animals per group with ± SEM. (B.) % of surviving animals during infection period. Source data are provided as a Source Data file.

Fig. S7

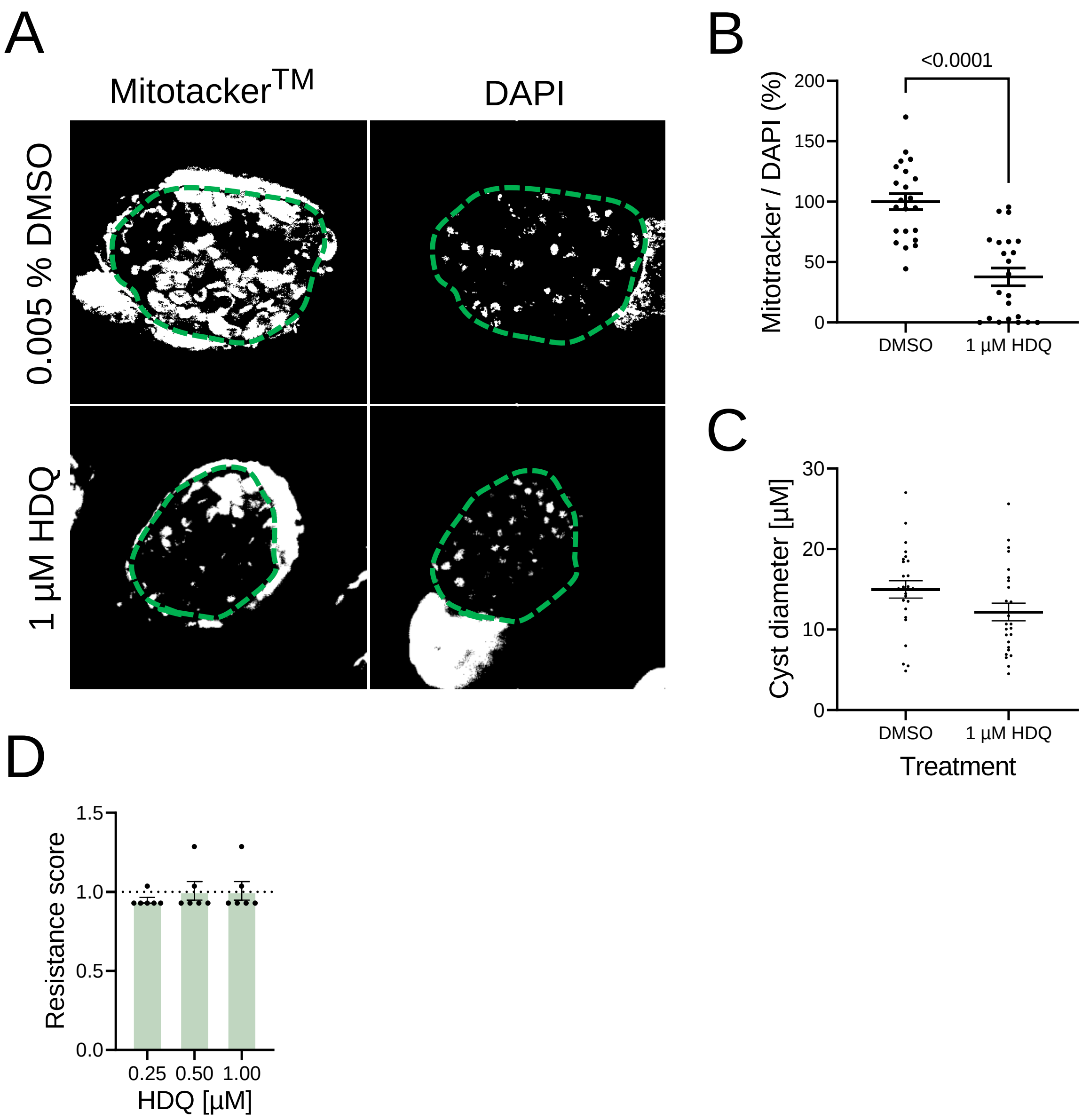

Figure S7: Impact of HDQ treatment on bradyzoite membrane potential and vitality. (A.-C.) Human KD3 myotubes were infected with ME49 tachyzoites under bradyzoite inducing conditions for 28 days, treated for seven days with either 0.005 % DMSO as solvent control or indicated concentration of HDQ and stained with Mitotracker^{TM} Deep Red, DBA and DAPI. Images of 22 cysts per group from two experimental replicates were randomly recorded at identical exposure times. DBA was used to determine the outline of the cysts (green dashed line). For determination of mitochondrial activity, images were converted into monochromatic images and thresholds were set for grey values as demonstrated in A (representative of two experiments). We normalized the Mitotracker^{TM} signal (23 to 255) by that of DAPI (19 to 255) per tissue cyst. Values are expressed as means and SEM from two independent experiments. ($p \leq 0.0001$, two-tailed Mann-Whitney-U-test, $p < 0.0001$). (C.) Mean diameters with SEM of 25 cysts from two independent experiments are shown. Scale bars indicate 10 µm. (D.) Resistance scores of 28-day-old Pru-tdTomato bradyzoites treated with indicated concentrations of HDQ or 0.005 % DMSO as solvent control. Shown are means and SEM of two independent experiments for bradyzoites performed in triplicates. Source data are provided as a Source Data file.

Fig. S8

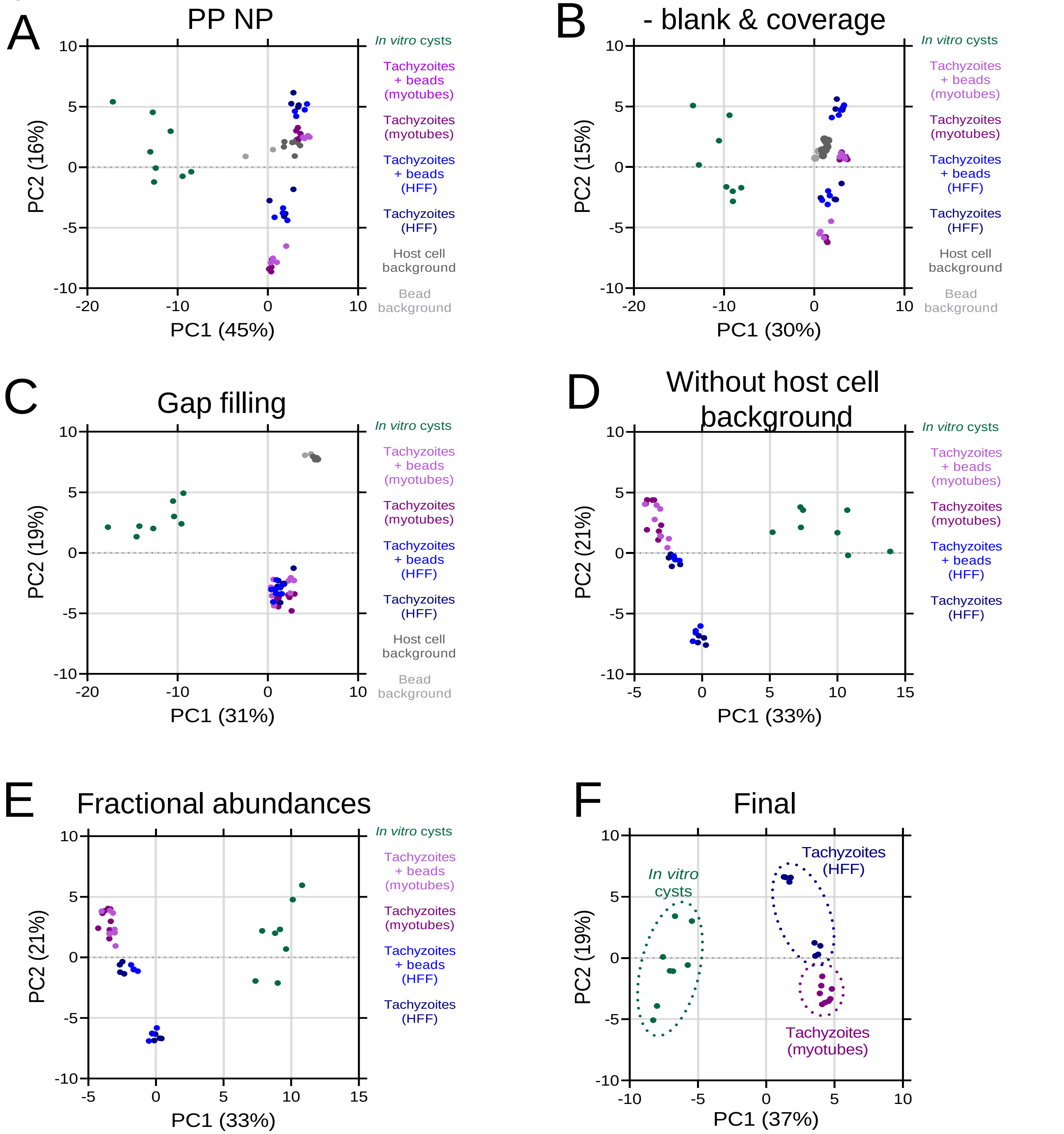

Figure S8: Data curation LC/MS. Samples were analyzed in positive and negative ionization mode separately. The principal component analyses illustrate effects of the data analysis steps. (A.) Positive and negative datasets were combined. (B.) Blanks were subtracted. Metabolites that had a coverage of less than 50 % in at least one sample set were excluded. (C.) Gaps in the dataset were filled with the mean intensity of the sample group of the respective batch. If a metabolite was not detected in sample group the minimal intensity value was used for gap filling. (D.) Metabolites that were prominent within the magnetic bead or host cell background samples were excluded. (E.) To allow statistical comparison of the cyst samples, which were not normalized to parasite numbers, with the tachyzoites samples, the fractional abundances were calculated by dividing each metabolite intensity by the sum of all intensities per sample. (F.) Because the addition of beads to the tachyzoite samples did not play an important role, these controls were omitted from further analysis. Data represent two independent experiments with four replicates each. Source data are provided as a Source Data file.

Fig. S9

Name	Gene ID	Acute infection (10 d.p.i.)	Chronic infection (28 d.p.i.)	chronic/acute
TgAT	TGME49_244440	75.64	3.5	0.05
TgAT1 high affinity	TGME49_233130	27.87	76.36	2.74
TgAT2 high affinity	TGME49_288540	30.4	36.33	1.20
TgApiAT5-3	TGME49_257530	1037.34	283.94	0.27
TgApiAT6-3	TGME49_249580	24.05	8.7	0.36
TgApiAT5-1	TGME49_248610	14.27	6.89	0.48
TgApiAT7-1	TGME49_263230	49.22	25.71	0.52
TgApiAT1	TGME49_215490	83.03	45.73	0.55
TgApiAT6-2	TGME49_290860	30.76	16.92	0.55
TgApiAT3-1	TGME49_318150	13.73	8.06	0.59
TgApiAT7-2	TGME49_263260	71.19	44.06	0.62
TgApiAT2	TGME49_320020	38.62	24.4	0.63
TgApiAT3-2	TGME49_248420	41.93	28.78	0.69
TgApiAT6-1	TGME49_240810	40.39	28.78	0.71
TgApiAT5-5	TGME49_293420	0.01	0.01	1.00
TgApiAT5-6	TGME49_293425	0.01	0.01	1.00
TgApiAT3-3	TGME49_220600	24.69	26.66	1.08
TgApiAT5-4	TGME49_216710	4.26	9.05	2.12
TgApiAT5-2	TGME49_205520	0.01	2.11	211.00

Figure S9: Transcriptomic data of amino acid transporter genes of *T. gondii* from Pittman et al., 2014[68]. Transcripts per kilobase million were downloaded from ToxoDB (http://www.toxodb.org) and analyzed for stage specific regulation by dividing expression values for acute (10 days post infection (d.p.i.)) by chronic infection in mice (28 d.p.i.).
