# Supplementary material for: In vitro maturation of Toxoplasma gondii bradyzoites in human myotubes and their metabolomic characterization
Source: Nat Commun. 2022 Mar 4;13:1168. doi: 10.1038/s41467-022-28730-w (PMC8897399; doi:10.1038/s41467-022-28730-w)
Supplement: Supplementary file 3 — Description of Supplementary Files [file 41467_2022_28730_MOESM3_ESM.pdf]

Supplementary Data 1
Description: Curation of extracted metabolite data. Significance calculations were done using an uncorrected two-tailed Mann-Whitney-U test.

Supplementary Data 2
Description: Curation of extracted metabolite data. Significance calculations were done using an uncorrected two-tailed Mann-Whitney-U test.

Supplementary Movie 1
Description: Twitching myotubes are shown in real time. Scale 20 µm.

Supplementary Movie 2
Description: Twitching myotubes are shown in real time. Scale 20 µm.
