# Supplementary material for: In vitro maturation of Toxoplasma gondii bradyzoites in human myotubes and their metabolomic characterization
Source: Nat Commun. 2022 Mar 4;13:1168. doi: 10.1038/s41467-022-28730-w (PMC8897399; doi:10.1038/s41467-022-28730-w)
Supplement: Supplementary file 8 — Reporting Summary [file 41467_2022_28730_MOESM8_ESM.pdf]

nature portfolio

Corresponding author(s): *Martin Blume*

Last updated by author(s): *Dec 20, 2021*

Reporting Summary

Nature Portfolio wishes to improve the reproducibility of the work that we publish. This form provides structure for consistency and transparency in reporting. For further information on Nature Portfolio policies, see our Editorial Policies and the Editorial Policy Checklist.

Statistics

For all statistical analyses, confirm that the following items are present in the figure legend, table legend, main text, or Methods section.

n/a	Confirmed	
☐	☒	The exact sample size (*n*) for each experimental group/condition, given as a discrete number and unit of measurement
☐	☒	A statement on whether measurements were taken from distinct samples or whether the same sample was measured repeatedly
☐	☒	The statistical test(s) used AND whether they are one- or two-sided<br>*Only common tests should be described solely by name; describe more complex techniques in the Methods section.*
☒	☐	A description of all covariates tested
☐	☒	A description of any assumptions or corrections, such as tests of normality and adjustment for multiple comparisons
☐	☒	A full description of the statistical parameters including central tendency (e.g. means) or other basic estimates (e.g. regression coefficient) AND variation (e.g. standard deviation) or associated estimates of uncertainty (e.g. confidence intervals)
☐	☒	For null hypothesis testing, the test statistic (e.g. *F*, *t*, *r*) with confidence intervals, effect sizes, degrees of freedom and *P* value noted<br>*Give P values as exact values whenever suitable.*
☒	☐	For Bayesian analysis, information on the choice of priors and Markov chain Monte Carlo settings
☒	☐	For hierarchical and complex designs, identification of the appropriate level for tests and full reporting of outcomes
☒	☐	Estimates of effect sizes (e.g. Cohen's *d*, Pearson's *r*), indicating how they were calculated

Our web collection on statistics for biologists contains articles on many of the points above.

Software and code

Policy information about availability of computer code

Data collection	*We used Compound Discoverer 3.1 (Thermo Fisher). We used Xcalibur v. 4.2.47 (Thermo Fisher) to record metabolomics data and Zen 2012 (Zeiss) to record maging data. Multiple Image Alignment, iTEM (EMSIS) v 5.2 (Build 3468) software was used for EM images. We used (i-control software (TECAN) v2.0.10.0 to record fluorescent intensities during growth assays.*
Data analysis	*We used the follwing tool to calculate the myogenic index of our KD3 myotube cells:<br>https://github.com/neumann-alexander/ccean<br>DOI ist 10.5281/zenodo.5528307<br><br>We used ClustVis 2.0 (https://biit.cs.ut.ee/clustvis/#editions) for PCA analysis of metabolomic data.<br><br>We used Excel 2019 (Microsoft), GraphPad Prism v8 and v9 for general data analysis and plotting.<br><br>We used Zen 2012 (Zeiss) for image analysis and ImageJ v1.52a for quantitative analyses of fluorescence images.<br><br>We used mzCloud database mirrored in mzVault (Thermo Fisher Scientic) v2.1.22.15 as of May 2020*

For manuscripts utilizing custom algorithms or software that are central to the research but not yet described in published literature, software must be made available to editors and reviewers. We strongly encourage code deposition in a community repository (e.g. GitHub). See the Nature Portfolio guidelines for submitting code & software for further information.

Data

Policy information about availability of data

All manuscripts must include a data availability statement. This statement should provide the following information, where applicable:

- Accession codes, unique identifiers, or web links for publicly available datasets
- A description of any restrictions on data availability
- For clinical datasets or third party data, please ensure that the statement adheres to our policy

Data is available as a supplement to this manuscript.

Field-specific reporting

Please select the one below that is the best fit for your research. If you are not sure, read the appropriate sections before making your selection.

☒ Life sciences ☐ Behavioural & social sciences ☐ Ecological, evolutionary & environmental sciences

For a reference copy of the document with all sections, see nature.com/documents/nr-reporting-summary-flat.pdf

Life sciences study design

All studies must disclose on these points even when the disclosure is negative.

Sample size

Sample sizes for LCMS experiments were determined by feasibility and limited by a calibration window of 72 hours of LCMS instrument. This sample size is sufficient to achieve statistical significance for the indicated metabolites and separation of sample types in multivariate analyses. Sample sizes in other experiments were choosen to reflect biological and technical variance of the investigated parameters based on past similar experiments.

Data exclusions

No data was excluded from analyses. Electron and fluorescent microscopy images are representative images of multiple. No images were omitted from quantifications.

Replication

Generally our results section and figure legends describe this replications. In particular, we performed the following replications:
Figure1:
Two independent experiments in technical triplicates.
Figure2:
Three independent experiments in technical triplicates.
Figure3B:
Total 9 measurements of three digestion reactions from one experiment (undigested controls represent two mock digestions).
Figure3C:
three independent experiments performed in triplicates.
Figure3D:
three or two independent experiments in triplicates.
Figure 4A:
One experiment and 50 measurements.
Figure 4B:
Data from two independent experiments with five mice per group.
Figure 4C:
One experiment with five mice per group.
Figure 5B:
One experiment with five technical replicates.
Figure 5C,D:
Three independent experiment with five technical replicates each.
Figure 6A-E, Figure SS8:
Two independent experiments with four replicates each.
Figure 7A:
Three independent experiments with three replicates each.
Figure 7B:
Two (Bradyzoites) and three independent experiments with three replicates each.
Figure 7C:
One experiment with four replicates for bradyzoite groups, five replicates for solvent treated and three replicates for NaFAc treated tachyzoites.
Figure 7D:
One experiment with four replicates for bradyzoite groups and three replicates for tachyzoite groups.
Video S1:
One experiment with one replicate.
Figure S1:
Representative images of multiple.

Figure S2:
One experiment, one replicate.
Figure S3:
One experiment with means of 25 measurements.
Figure S4:
One experiment, one replicate.
Figure S5:
One experiment, one replicate.
Figure S6A:
Two experiments with five replicate mouse infections each.
Figure S6B:
Two experiments with five replicate mouse infections each, except for one experiment that had 6 replicate HFF-derived tachyzoite infection.
Figure S7A:
One experiment, one replicate.
Figure S7B:
Two independent experiments with 22 replicate measurements in total.
Figure S7C:
Two independent experiments with 25 replicate measurements in total.

All repetitions were successful.

Randomization	LCMS samples were randomized before analysis, except for blanks. Mice were randomly assigned to experimental groups. Covariates in 96well plate based gowth assays experiments was excluded by independent repetitions of all experiments using parasites and host cells of different passages and different plate layouts. Data in Fig S3 represent one sample per condition. No statistical analysis was conducted in this case. Experiments done to illustrate primary data were performed as single experiments.
Blinding	Generally, we did not blind our samples, if not explictly mentioned. In particular, our LCMS data analysis requires the identities of samples. We needed to know which samples were uninfected and needed to be subtracted as background. We also needed to know controls for quenching procedures that were omitted for final analysis for fold-change calculations. The procedure is detailed in the results and methods sections.

Behavioural & social sciences study design

All studies must disclose on these points even when the disclosure is negative.

Study description	Briefly describe the study type including whether data are quantitative, qualitative, or mixed-methods (e.g. qualitative cross-sectional, quantitative experimental, mixed-methods case study).
Research sample	State the research sample (e.g. Harvard university undergraduates, villagers in rural India) and provide relevant demographic information (e.g. age, sex) and indicate whether the sample is representative. Provide a rationale for the study sample chosen. For studies involving existing datasets, please describe the dataset and source.
Sampling strategy	Describe the sampling procedure (e.g. random, snowball, stratified, convenience). Describe the statistical methods that were used to predetermine sample size OR if no sample-size calculation was performed, describe how sample sizes were chosen and provide a rationale for why these sample sizes are sufficient. For qualitative data, please indicate whether data saturation was considered, and what criteria were used to decide that no further sampling was needed.
Data collection	Provide details about the data collection procedure, including the instruments or devices used to record the data (e.g. pen and paper, computer, eye tracker, video or audio equipment) whether anyone was present besides the participant(s) and the researcher, and whether the researcher was blind to experimental condition and/or the study hypothesis during data collection.
Timing	Indicate the start and stop dates of data collection. If there is a gap between collection periods, state the dates for each sample cohort.
Data exclusions	If no data were excluded from the analyses, state so OR if data were excluded, provide the exact number of exclusions and the rationale behind them, indicating whether exclusion criteria were pre-established.
Non-participation	State how many participants dropped out/declined participation and the reason(s) given OR provide response rate OR state that no participants dropped out/declined participation.
Randomization	If participants were not allocated into experimental groups, state so OR describe how participants were allocated to groups, and if allocation was not random, describe how covariates were controlled.

Ecological, evolutionary & environmental sciences study design

All studies must disclose on these points even when the disclosure is negative.

Study description	*Briefly describe the study. For quantitative data include treatment factors and interactions, design structure (e.g. factorial, nested, hierarchical), nature and number of experimental units and replicates.*
Research sample	*Describe the research sample (e.g. a group of tagged Passer domesticus, all Stenocereus thurberi within Organ Pipe Cactus National Monument), and provide a rationale for the sample choice. When relevant, describe the organism taxa, source, sex, age range and any manipulations. State what population the sample is meant to represent when applicable. For studies involving existing datasets, describe the data and its source.*
Sampling strategy	*Note the sampling procedure. Describe the statistical methods that were used to predetermine sample size OR if no sample-size calculation was performed, describe how sample sizes were chosen and provide a rationale for why these sample sizes are sufficient.*
Data collection	*Describe the data collection procedure, including who recorded the data and how.*
Timing and spatial scale	*Indicate the start and stop dates of data collection, noting the frequency and periodicity of sampling and providing a rationale for these choices. If there is a gap between collection periods, state the dates for each sample cohort. Specify the spatial scale from which the data are taken*
Data exclusions	*If no data were excluded from the analyses, state so OR if data were excluded, describe the exclusions and the rationale behind them, indicating whether exclusion criteria were pre-established.*
Reproducibility	*Describe the measures taken to verify the reproducibility of experimental findings. For each experiment, note whether any attempts to repeat the experiment failed OR state that all attempts to repeat the experiment were successful.*
Randomization	*Describe how samples/organisms/participants were allocated into groups. If allocation was not random, describe how covariates were controlled. If this is not relevant to your study, explain why.*
Blinding	*Describe the extent of blinding used during data acquisition and analysis. If blinding was not possible, describe why OR explain why blinding was not relevant to your study.*

Did the study involve field work? ☐ Yes ☐ No

Field work, collection and transport

Field conditions	*Describe the study conditions for field work, providing relevant parameters (e.g. temperature, rainfall).*
Location	*State the location of the sampling or experiment, providing relevant parameters (e.g. latitude and longitude, elevation, water depth).*
Access & import/export	*Describe the efforts you have made to access habitats and to collect and import/export your samples in a responsible manner and in compliance with local, national and international laws, noting any permits that were obtained (give the name of the issuing authority, the date of issue, and any identifying information).*
Disturbance	*Describe any disturbance caused by the study and how it was minimized.*

Reporting for specific materials, systems and methods

We require information from authors about some types of materials, experimental systems and methods used in many studies. Here, indicate whether each material, system or method listed is relevant to your study. If you are not sure if a list item applies to your research, read the appropriate section before selecting a response.

Materials & experimental systems

n/a	Involved in the study	
☐	☒	Antibodies
☐	☒	Eukaryotic cell lines
☒	☐	Palaeontology and archaeology
☐	☒	Animals and other organisms
☒	☐	Human research participants
☒	☐	Clinical data
☒	☐	Dual use research of concern

Methods

n/a	Involved in the study	
☒	☐	ChIP-seq
☒	☐	Flow cytometry
☒	☐	MRI-based neuroimaging

Antibodies

Antibodies used	*anti-CC2 (non-commercial, obtained as a gift from Carsten Lueder, University of Göttingen, Germany, used at 1:1000 dilution), DG52 mab anti-Sag1 (non-commercial, obtained as a gift from David Sibley, Washington University, Missouri, US, used at 1:500 dilution) in St. Louis), myosin heavy chain MF20 (ebioscience, Cat-no: 14-6503-82, Lot 4301341, used at 1:400 dilution); streptavidin-Cy5 (Jackson ImmunoResearch, Cat: 016-170-084, Lot: 151872, used at 1:2500 dilution); goat anti-rat-Alexa546 (Invitrogen, Cat: A-11081, Lot: 2146039, used at 1:300 dilution), rabbit anti-mouse-Cy3 (Dianova, Cat: 315-165-003, Lot: 120198, used at 1:400 dilution), Streptavidin-Alexa488 (Invitrogen, Cat: S32354, Lot from 2009, used at 1:2500 dilution), donkey anti-mouse-Cy5 (Dianova, Cat: 715-175-151, unknown Lot, used at 1:300 dilution), goat anti-rat-Alexa350 (Thermo Fisher Scientific, A-21093, Lot:2017373, used at 1:300 dilution)*
Validation	*anti-cc2: Gross, U. et al. Monoclonal rat antibodies directed against Toxoplasma gondii suitable for studying tachyzoite-bradyzoite interconversion in vivo. Clin Diagn Lab Immunol 2, 542-548 (1995).*<br>*antiSag1: Bulow, R. & Boothroyd, J. C. Protection of mice from fatal Toxoplasma gondii infection by immunization with p30 antigen in liposomes. J Immunol 147, 3496-3500 (1991).*<br>*MF20: This Antibody was verified by Cell treatment to ensure that the antibody binds to the antigen stated. Myosin 4 Antibody*<br>*Other antibodies were validated as per manufacturers specifications.*

Eukaryotic cell lines

Policy information about cell lines

Cell line source(s)	*immortalized human myoblast cell line KD3 N. Hashimoto, doi:10.1038/gt.2011.44 (2011)*<br>*human foreskin fibroblasts (HFF): BJ-5ta ATCC CRL-.4001*<br>*RH and VEG parasites were a kind gift of Gereon Schares, Friedrich Loeffler Institute*<br>*Pru-tdTomato was a kind gift from Boris Striepen DOI:10.1016/j.immuni.2008.07.012 (2008)*<br>*ME49, NTE parasites were a kind gift of Uwe Gross DOI: 10.1007/s004360050280 (1997)*<br>*Pru-GFP parasites were sourced from David Bzik: DOI: 10.1128/EC.00297-10 (2011)*
Authentication	*Cells lines were authenticated by visual inspection of morphology (KD3 and HFF) or their flourescence Toxoplasma Pru-tdTomato, Pru-GFP. Other Toxoplasma cell lines were not been authenticated.*
Mycoplasma contamination	*All cell lines tested negative for Mycoplasma.*
Commonly misidentified lines (See ICLAC register)	*According to ILCA the register (release v11) no commonly misidentified cell lines were used in this study.*

Palaeontology and Archaeology

Specimen provenance	*Provide provenance information for specimens and describe permits that were obtained for the work (including the name of the issuing authority, the date of issue, and any identifying information). Permits should encompass collection and, where applicable, export.*
Specimen deposition	*Indicate where the specimens have been deposited to permit free access by other researchers.*
Dating methods	*If new dates are provided, describe how they were obtained (e.g. collection, storage, sample pretreatment and measurement), where they were obtained (i.e. lab name), the calibration program and the protocol for quality assurance OR state that no new dates are provided.*

☐ Tick this box to confirm that the raw and calibrated dates are available in the paper or in Supplementary Information.

Ethics oversight	*Identify the organization(s) that approved or provided guidance on the study protocol, OR state that no ethical approval or guidance was required and explain why not.*

Note that full information on the approval of the study protocol must also be provided in the manuscript.

Animals and other organisms

Policy information about studies involving animals; ARRIVE guidelines recommended for reporting animal research

Laboratory animals	*10-week-old male and female mice (C57BL/6JRj) were used in this study.*
Wild animals	*No wild animals were used in this study.*
Field-collected samples	*No field cohort samples were used in this study.*
Ethics oversight	*Experiments were approved by the state of Baden-Württemberg (Regierungspräsidium Freiburg; reference number 35-9185.81/ G-19/89).*

Note that full information on the approval of the study protocol must also be provided in the manuscript.

Human research participants

Policy information about studies involving human research participants

Population characteristics	*Describe the covariate-relevant population characteristics of the human research participants (e.g. age, gender, genotypic information, past and current diagnosis and treatment categories). If you filled out the behavioural & social sciences study design questions and have nothing to add here, write "See above."*
Recruitment	*Describe how participants were recruited. Outline any potential self-selection bias or other biases that may be present and how these are likely to impact results.*
Ethics oversight	*Identify the organization(s) that approved the study protocol.*

Note that full information on the approval of the study protocol must also be provided in the manuscript.

Clinical data

Policy information about clinical studies

All manuscripts should comply with the ICMJEguidelines for publication of clinical research and a completedCONSORT checklist must be included with all submissions.

Clinical trial registration	*Provide the trial registration number from ClinicalTrials.gov or an equivalent agency.*
Study protocol	*Note where the full trial protocol can be accessed OR if not available, explain why.*
Data collection	*Describe the settings and locales of data collection, noting the time periods of recruitment and data collection.*
Outcomes	*Describe how you pre-defined primary and secondary outcome measures and how you assessed these measures.*

Dual use research of concern

Policy information about dual use research of concern

Hazards

Could the accidental, deliberate or reckless misuse of agents or technologies generated in the work, or the application of information presented in the manuscript, pose a threat to:

No	Yes	
☐	☐	Public health
☐	☐	National security
☐	☐	Crops and/or livestock
☐	☐	Ecosystems
☐	☐	Any other significant area

Experiments of concern

Does the work involve any of these experiments of concern:

No	Yes	
☐	☐	Demonstrate how to render a vaccine ineffective
☐	☐	Confer resistance to therapeutically useful antibiotics or antiviral agents
☐	☐	Enhance the virulence of a pathogen or render a nonpathogen virulent
☐	☐	Increase transmissibility of a pathogen
☐	☐	Alter the host range of a pathogen
☐	☐	Enable evasion of diagnostic/detection modalities
☐	☐	Enable the weaponization of a biological agent or toxin
☐	☐	Any other potentially harmful combination of experiments and agents

ChIP-seq

Data deposition

☐ Confirm that both raw and final processed data have been deposited in a public database such as GEO.

☐ Confirm that you have deposited or provided access to graph files (e.g. BED files) for the called peaks.

Data access links<br>*May remain private before publication.*	*For "Initial submission" or "Revised version" documents, provide reviewer access links. For your "Final submission" document, provide a link to the deposited data.*
Files in database submission	*Provide a list of all files available in the database submission.*
Genome browser session<br>(e.g. UCSC)	*Provide a link to an anonymized genome browser session for "Initial submission" and "Revised version" documents only, to enable peer review. Write "no longer applicable" for "Final submission" documents.*

Methodology

Replicates	*Describe the experimental replicates, specifying number, type and replicate agreement.*
Sequencing depth	*Describe the sequencing depth for each experiment, providing the total number of reads, uniquely mapped reads, length of reads and whether they were paired- or single-end.*
Antibodies	*Describe the antibodies used for the ChIP-seq experiments; as applicable, provide supplier name, catalog number, clone name, and lot number.*
Peak calling parameters	*Specify the command line program and parameters used for read mapping and peak calling, including the ChIP, control and index files used.*
Data quality	*Describe the methods used to ensure data quality in full detail, including how many peaks are at FDR 5% and above 5-fold enrichment.*
Software	*Describe the software used to collect and analyze the ChIP-seq data. For custom code that has been deposited into a community repository, provide accession details.*

Flow Cytometry

Plots

Confirm that:

- [ ] The axis labels state the marker and fluorochrome used (e.g. CD4-FITC).
- [ ] The axis scales are clearly visible. Include numbers along axes only for bottom left plot of group (a 'group' is an analysis of identical markers).
- [ ] All plots are contour plots with outliers or pseudocolor plots.
- [ ] A numerical value for number of cells or percentage (with statistics) is provided.

Methodology

Sample preparation	*Describe the sample preparation, detailing the biological source of the cells and any tissue processing steps used.*
Instrument	*Identify the instrument used for data collection, specifying make and model number.*
Software	*Describe the software used to collect and analyze the flow cytometry data. For custom code that has been deposited into a community repository, provide accession details.*
Cell population abundance	*Describe the abundance of the relevant cell populations within post-sort fractions, providing details on the purity of the samples and how it was determined.*
Gating strategy	*Describe the gating strategy used for all relevant experiments, specifying the preliminary FSC/SSC gates of the starting cell population, indicating where boundaries between "positive" and "negative" staining cell populations are defined.*

- [ ] Tick this box to confirm that a figure exemplifying the gating strategy is provided in the Supplementary Information.

Magnetic resonance imaging

Experimental design

Design type	*Indicate task or resting state; event-related or block design.*
Design specifications	*Specify the number of blocks, trials or experimental units per session and/or subject, and specify the length of each trial or block (if trials are blocked) and interval between trials.*
Behavioral performance measures	*State number and/or type of variables recorded (e.g. correct button press, response time) and what statistics were used to establish that the subjects were performing the task as expected (e.g. mean, range, and/or standard deviation across subjects).*

Acquisition

Imaging type(s) | *Specify: functional, structural, diffusion, perfusion.*

Field strength | *Specify in Tesla*

Sequence & imaging parameters | *Specify the pulse sequence type (gradient echo, spin echo, etc.), imaging type (EPI, spiral, etc.), field of view, matrix size, slice thickness, orientation and TE/TR/flip angle.*

Area of acquisition | *State whether a whole brain scan was used OR define the area of acquisition, describing how the region was determined.*

Diffusion MRI ☐ Used ☐ Not used

Preprocessing

Preprocessing software | *Provide detail on software version and revision number and on specific parameters (model/functions, brain extraction, segmentation, smoothing kernel size, etc.).*

Normalization | *If data were normalized/standardized, describe the approach(es): specify linear or non-linear and define image types used for transformation OR indicate that data were not normalized and explain rationale for lack of normalization.*

Normalization template | *Describe the template used for normalization/transformation, specifying subject space or group standardized space (e.g. original Talairach, MNI305, ICBM152) OR indicate that the data were not normalized.*

Noise and artifact removal | *Describe your procedure(s) for artifact and structured noise removal, specifying motion parameters, tissue signals and physiological signals (heart rate, respiration).*

Volume censoring | *Define your software and/or method and criteria for volume censoring, and state the extent of such censoring.*

Statistical modeling & inference

Model type and settings | *Specify type (mass univariate, multivariate, RSA, predictive, etc.) and describe essential details of the model at the first and second levels (e.g. fixed, random or mixed effects; drift or auto-correlation).*

Effect(s) tested | *Define precise effect in terms of the task or stimulus conditions instead of psychological concepts and indicate whether ANOVA or factorial designs were used.*

Specify type of analysis: ☐ Whole brain ☐ ROI-based ☐ Both

Statistic type for inference
(See Eklund et al. 2016) | *Specify voxel-wise or cluster-wise and report all relevant parameters for cluster-wise methods.*

Correction | *Describe the type of correction and how it is obtained for multiple comparisons (e.g. FWE, FDR, permutation or Monte Carlo).*

Models & analysis

n/a	Involved in the study
☐	☐ Functional and/or effective connectivity
☐	☐ Graph analysis
☐	☐ Multivariate modeling or predictive analysis

Functional and/or effective connectivity | *Report the measures of dependence used and the model details (e.g. Pearson correlation, partial correlation, mutual information).*

Graph analysis | *Report the dependent variable and connectivity measure, specifying weighted graph or binarized graph, subject- or group-level, and the global and/or node summaries used (e.g. clustering coefficient, efficiency, etc.).*

Multivariate modeling and predictive analysis | *Specify independent variables, features extraction and dimension reduction, model, training and evaluation metrics.*
